# New specimens of *Bunaia woodwardi* Clarke, 1919 (Euchelicerata): a new member of Offacolidae providing insight supporting the Arachnomorpha

**DOI:** 10.1098/rsos.240499

**Published:** 2024-10-30

**Authors:** Lorenzo Lustri, Jonathan B. Antcliffe, Pierre Gueriau, Allison C. Daley

**Affiliations:** ^1^ Institute of Earth Sciences, University of Lausanne, Géopolis, Lausanne CH-1015, Switzerland; ^2^ Université Paris-Saclay, CNRS, ministère de la Culture, UVSQ, MNHN, Institut photonique d’analyse non-destructive européen des matériaux anciens, Saint-Aubin 91192, France

**Keywords:** Offacolidae, Euchelicerata, Arachnomorpha, Artiopoda, Vicissicaudata, synziphosurine

## Abstract

The rapid early diversification of arthropods has made understanding internal relationships within the group fiendish. Particularly unresolved is the origin of Euchelicerata, a clade consisting of the Prosomapoda (comprising the extant Xiphosura and Arachnida and the extinct Chasmataspidida, Eurypterida and synziphosurines) and the extinct Offacolidae. Here we describe new material of the Silurian ‘synziphosurine’ *Bunaia woodwardi* Clarke, 1919 that reveals previously unknown features of its ventral anatomy: a pair of elongated chelicerae in the prosoma, followed posteriorly by five pairs of biramous appendages, a first pre-abdomen somite bearing a pair of paddle-like uniramous appendages (exopods) and a ventral pretelsonic process. Phylogenetic analyses retrieve *B. woodwardi* as a member of Offacolidae closely related to *Setapedites abundantis* from the Early Ordovician Fezouata Biota. An anatomical comparison of the pretelsonic process of *B. woodwardi*, also present in *Setapedites*, with the posterior trunk morphologies of other Offacolidae, Habeliida and Vicissicaudata, suggests a possible homologous appendicular origin. This proposed apomorphic character supports a monophyletic Arachnomorpha, formed of Vicissicaudata, Habeliida and Euchelicerata. The establishment of this new homology could help to clarify the highly enigmatic phylogeny at the base of the euchelicerates as well as the sequence of character acquisition during their early evolution.

## 1. Introduction

Arthropoda is divided into several major groupings whose relationships are highly controversial and subject to rapid revision and terminological changes [1–7]. One such major division within the arthropods is Euchelicerata, a highly diverse clade that includes spiders, scorpions and mites (arachnids), as well as horseshoe crabs (xiphosurans). Euchelicerata also contains several important extinct forms such as sea scorpions (eurypterids), chasmataspidids and synziphosurines. The enigmatic synziphosurines, which superficially resemble modern horseshoe crabs, dwelt in the seas from the Early Ordovician (Tremadocian (*ca* 484 Ma) [8] to the Late Carboniferous (Mississippian (*ca* 323 Ma)) [9]. They presumably scratched out a living searching for food on the sediment surface of these ancient seabeds and may in some species have had a limited swimming ability, rather like modern horseshoe crabs.

Though the phylogenetic position of these synziphosurines has proven problematic for decades, they may yet prove essential for understanding the earliest evolution of the Euchelicerata. Once considered part of the monophyletic Xiphosura [10], the synziphosurines have also been considered paraphyletic [11] or even polyphyletic [12]. The polyphyletic reinterpretation fractures the group with some taxa placed as basal to all other euchelicerates, while others were scattered around the euchelicerate tree, as sister taxa of chasmataspids, eurypterids and arachnids. In this framework, the exceptionally well-preserved synziphosurines *Dibasterium durgae* [13] and *Offacolus kingi* [14,15] have been considered to be stem-euchelicerates, possessing defining characters of the modern euchelicerates such as gill opercula and chelicerae. The presence of biramous appendages in the prosoma of *Dibasterium* and *Offacolus* led to the establishment of the clade Prosomapoda, which is the clade of euchelicerates defined by uniramous prosomal appendages [12]. The discovery and description of *Setapedites abundantis* [8] from the Early Ordovician Fezouata Biota [16,17] united *Dibasterium*, *Offacolus* and *Setapedites* itself into the family Offacolidae [15]. *Habelia optata* [18], previously proposed as the closest Cambrian relative to the euchelicerates [19], was further supported in this position, on the basis of characters that the Cambrian taxon shares with the expanded Offacolidae (e.g. stenopodous prosomal exopods, uniramous first pair of appendages and bipartite opisthosomal tergites) [8,19]. Despite the resolved position of Offacolidae, questions remain about certain anatomical characters in *Setapedites*, particularly about the nature of its pretelsonic process [8]. A possible comparable structure was described by Simonetta [20] in *H. optata* as a para-anal lamina (in Italian, ‘lamina paraanale’) closely related to the post-ventral plate of *Aglaspis spinifer* [21], but Whittington [22] refuted this hypothesis, interpreting this feature as a taphonomic artefact. New material then allowed Aria & Caron [19] to confirm the presence of a rounded plate below the telson head, which they interpreted as an anal pouch. On the basis of the pretelsonic process [8], a potential relationship to the Vicissicaudata [23] (the clade including *Emeraldella* [24], *Sidneyia* [18], the Aglaspidida [18] and the Cheloniellida [25]) has also been hypothesized for *Setapedites*. A better understanding of synziphosurine anatomy is essential to reconstructing the relationships not only within Euchelicerata but also between euchelicerates and their potential relatives, Habeliida and Vicissicaudata.

New specimens assigned to the synziphosurine species *Bunaia woodwardi* Clarke, 1919 [26] from the late Silurian Bertie Lagerstätte (Ontario, Canada) are described herein. These specimens reveal details in their appendages and pretelsonic process. *Bunaia woodwardi* is the youngest offacolid described so far, extending the range of the clade up into the late Silurian and increasing the number of taxa in the clade to four. Detailed preservation of the pretelsonic process allows us to better constrain this character in the Offacolidae and its relation to the anal pouch of *H. optata* and the appendicular derivatives of Vicissicaudata. Vicissicaudata and Euchelicerata have been suggested to be closely related and to form the clade Arachnomorpha (*sensu* Størmer [27]), which has been retrieved as monophyletic by several phylogenetic analyses [4,19,28,29]. This study describes a possible homologous origin for the posterior trunk morphology of Vicissicaudata, Euchelicerata and Habeliida, providing support for a phylogenetic relationship among them and therefore supporting the broader Arachnomorpha hypothesis [27], rather than a monophyletic Artiopoda [30] (Trilobita and the Vicissicaudata), which does not include Euchelicerata and its sister group Habeliida.

## 2. Material and methods

### 2.1. Studied material and geological setting

Four new specimens were examined as part of this study. All the specimens are housed at the Royal Ontario Museum (ROM; catalogued as ROMIP53886, ROMIP51447, ROMIP50689 and ROMIP50690). They were collected from two quarries in the Williamsville Formation of the Bertie Group (Přídolí), located near Fort Erie, Ontario, Canada. The Bertie Group is composed of limestones, dolomitic limestones and evaporates, and outcrops are present in western New York State (US) and southeastern Ontario (Canada). The Williamsville is the Bertie Group’s upper formation, and it consists of fine-grained, argillaceous dolomite of different shades of grey and dolomitic mudstone [31–34]. The age of the formation is based on correlations with the unit below and above since the biostratigraphic control for this formation is poor [35].

Specimens ROMIP53886 and ROMIP51447 were collected from the Ridgemount Quarry (42°92′96″ N, 79°00′48″ W), while specimens ROMIP50689 and ROMIP50690 were collected from the George C. Campbell Quarry, approximately 1 mile south of Ridgemount Quarry.

These four new specimens were compared with new photographs (images credited to Russell Bicknell) of the original four specimens of *B. woodwardi* (catalogued as NYSM 9909, NYSM 9910, NYSM 9911 and NYSM 9912). Clarke [26] refers to this material as originating from the ‘Bertie Waterlime of the Salina Group’ from an outcrop at East of Buffalo (New York State, US). However, the following works recognized the Bertie and the Salina as different groups [31–34], so we attribute Clark’s original material to the Bertie Group.

### 2.2. Systematic framework

This work follows the systematics of refs. [4,8,12,19,23,36] and uses the anatomical terms presented in refs. [8,12,13,15,19,23,36,37].

### 2.3. Photography and multi-spectral imaging

**Figure 1.**
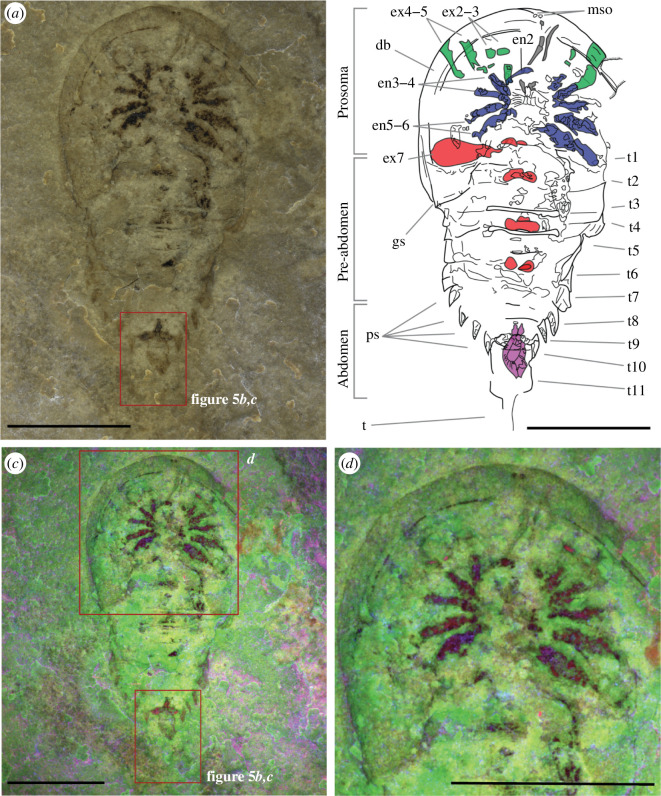
New specimen of *B. woodwardi* ROMIP53886. (*a*) Optical photograph under polarized filter and submerged in alcohol. (*b*) Interpretative line drawing. Highlighted in grey are the chelicerae, in green prosomal exopods, in blue endopods, in red opisthosomal exopods and in purple the pretelsonic process. (*c*) Multi-spectral imaging. (*d*) Multi-spectral imaging close-up from the upper boxed area in (*c*). Abbreviations: en2−5, endopods 2−5; ex2−6, exopods 2−6; mso, median sensory organs; ps, pleural spine; t1–t11, opisthosomal tergites 1−11; t, telson. Scale bars = 1 cm.

**Figure 2.**
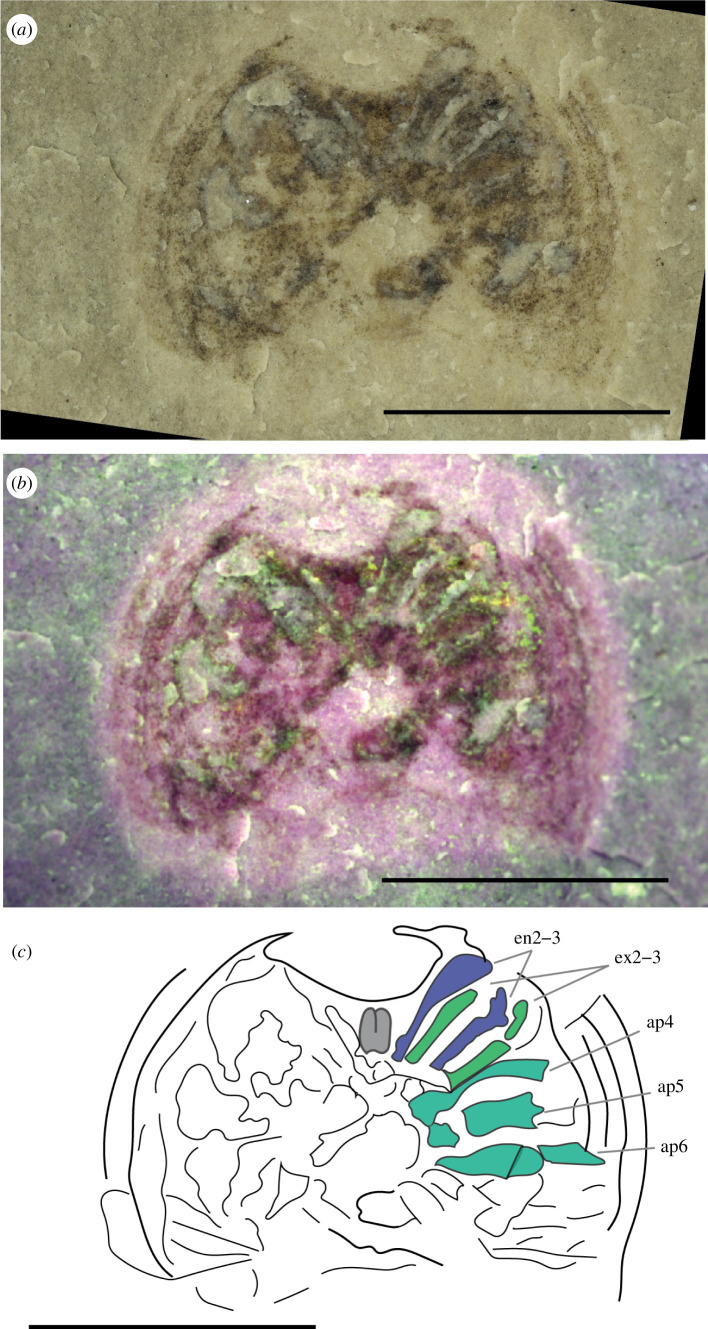
New specimen of *B. woodwardi* ROMIP51447. (*a*) Normal light optical photograph. (*b*) Multi-spectral imaging. (*c*) Interpretative line drawing. Highlighted in grey are the chelicera, in green prosomal exopods, in blue endopods and in aquamarine unidentified exopods or endopods of appendages 4−6. Abbreviations: ap4−6, appendages 4−6; en2−3, endopods 2−3; ex2−3, exopods 2−3. Scale bars = 5 mm.

**Figure 3.**
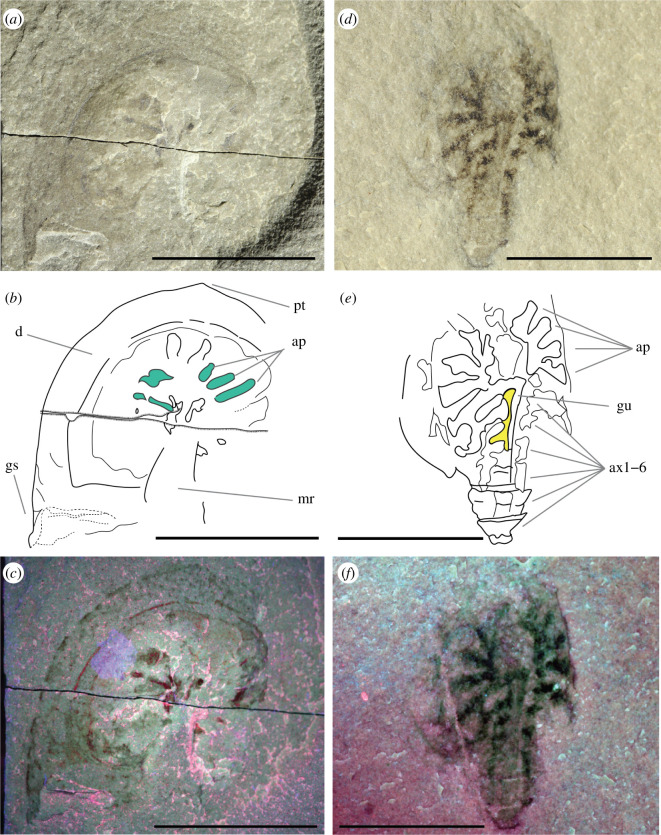
New specimens of *B. woodwardi* ROMIP50689 and ROMIP50690. (*a–c*) Normal light optical photograph (*a*), interpretative line drawing (*b*) and multi-spectral imaging (*c*) of specimen ROMIP50689. (*d–f*) Normal light optical photograph (*d*), interpretative line drawing (*e*) and multi-spectral imaging (*f*) of specimen ROMIP50690. Highlighted in aquamarine in (*b*) are unidentified exopods or endopods of the appendages, and in yellow, in (*e*) are remains of the digestive system. Abbreviations: ap, appendages; ax1−6, axis 1−6 of the opisthosomal tergites; d, doublure; gs, genal spine; gu, gut; mr, cardiac (median) ridge; tp, apical tip. Scale bars = 1 cm (*a–c*), 5 mm (*d–f*).

The specimens were each examined with a light microscope (Wild Heerbrugg M8) under normal light. Subsequently, they were photographed with an SLR camera (Canon EOS 800D equipped with CANON macro lens MP-E 65 mm 1:2.81−5×) mounted on a stand and connected to a z-stacking system (STACKSHOT 3X), using different combinations of lighting conditions: normal light, polarized light, dry, covered in alcohol. Z-stacks were rendered using the Helicon Focus software. All specimens were analysed in the Optical Laboratory at Lausanne University (Geopolis-3439). The specimens were also examined under multi-spectral macroimaging using an innovative set-up that allows for the collection of reflection and luminescence images in various spectral ranges from ultraviolet (UV) to near infrared domains [38–40]. The set-up is made of a low-noise 2.58-megapixel back-illuminated sCMOS camera (PRIME 95B 25 mm, Photometrics) with high sensitivity from 200 to 1000 nm, fitted with a UV–VIS–IR 60 mm 1:4 Apo macro lens (CoastalOptics) opposed by a filter wheel holding eight interference band-pass (Semrock) to collect images in eight different spectral ranges from 435 to 935 nm. Illumination with wavelengths ranging from 365 up to 700 nm was provided by 16 LEDs (CoolLED pE-4000), coupled to a liquid light-guide fibre fitted with a fibre-optic ring light-guide [38–40]. This set-up consequently allows for collecting images in a total of more than 90 different illumination/detection configurations. The resulting 16-bit greyscale images were combined into false colour red, green and blue (RGB) overlays in ImageJ, manually adjusting the minimum and maximum values for each image in order to provide the strongest possible enhancement of contrast, and thus reveal new details of the morphology of the specimens examined [38–40]. The false colour RGB overlays associated with each of the specimens analysed were produced using the following configurations: (figure 1*c,d*) red—illumination 525 nm/detection 571 ± 36 nm (reflection), green—illumination 470 nm/detection 719 ± 30 nm (luminescence), blue—illumination 385 nm/detection 515 ± 15 nm (luminescence); (figure 2*b*) red—illumination 580 nm/detection 935 ± 85 nm (luminescence), green—illumination 525 nm/detection 571 ± 36 nm (refl.), blue—illumination 470 nm/detection 719 ± 30 nm (luminescence); (figure 3*c*) red—illumination 525 nm/detection 571 ± 36 nm (refl.), green—illumination 470 nm/detection 719 ± 30 nm (luminescence), blue—illumination 365 nm/detection 571 ± 36 nm (luminescence); (figure 3*f*) red—illumination 365 nm/detection 571 ± 36 nm (luminescence), green—illumination 770 nm/detection 775 ± 23 nm (reflection), blue—illumination 525 nm/detection 571 ± 36 nm (reflection).

### 2.4. Interpretative drawings

Interpretative drawings were made in Adobe Illustrator directly from the photos, using a Wacom v. 6.3.38-3 graphic table and a pen tool.

### 2.5. Phylogenetic analyses

Parsimony and Bayesian analyses were performed using the recent standard methodology for this group [8,12]. *Bunaia woodwardi* was added to the expanded matrix from Lamsdell [12] used in Lustri *et al*. [8]. Character coding for *B. woodwardi* is available in the electronic supplementary material dataset. Parsimony analyses were run on TNT v. 1.5, using a traditional search with 100 000 random addition sequences followed by branch swapping with 100 000 repetitions. All characters were unordered and both equal weights followed by Bremer support (10 000 repetitions) and implied weight (3*K*, 6*K*, 9*K* and 12*K*) followed by relative Bremer support (10 000 repetitions) were applied. Bremer support was calculated on the strict consensus tree. Bayesian analyses were run on mrBayes v. 3.2.7a using a tree search followed an Mkv + Γ model [41] with four chains sampling during four runs for 10 000 000 Markov chain Monte Carlo generations, a tree sampled every 1000 generations and burn-in of 20%. A 50% majority rule tree and a strict consensus tree were both calculated. The mrBayes script is available as an electronic supplementary material dataset.

### 2.6. Abbreviations

#### 2.6.1. Institutional abbreviations

NIGPAS, Nanjing Institute of Geology and Palaeontology, China; NYSM, New York State Museum, USA; MPM, Milwaukee Public Museum, USA; ROMIP, Royal Ontario Museum Invertebrate Paleontology collections, Canada; UMNH, Natural History Museum of Utah, USA.

#### 2.6.2. Abbreviations used to label figures

ap, appendages; ax, axis; bf, bifurcation; ch, chelicera; mr, median ridge (cardiac ridge); cn, centrum; d, doublure; en2−5, endopods 2−5; ex2−6, exopods 2−6; gs, genal spine; gu, gut; in, insertion; mso, median sensory organs; pd, pleural spine t, telson; t1–t11, opisthosomal tergites 1−11.

## 3. Results

### 3.1. Systematic palaeontology

Arthropoda von Siebold, 1848.

Arachnomorpha Størmer, 1944.

Euchelicerata Weygoldt & Paulus, 1979.

Offacolidae Sutton, Briggs, Siveter, Siveter and Orr, 2002.

*Bunaia woodwardi* Clarke, 1919.

. 1919 *Bunaia woodwardi* Clarke, pp. 531−532, pl.14, figs. 1–4;

**Figure 4.**
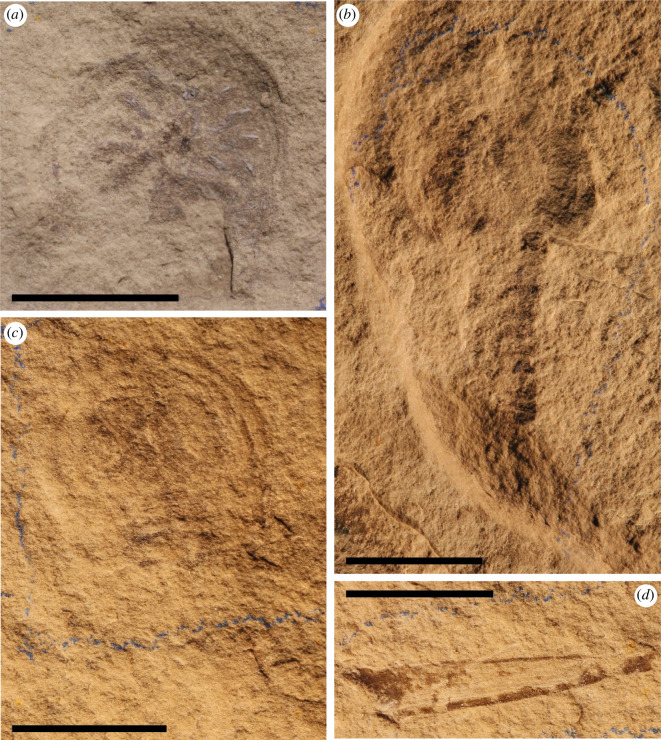
Lectotype and paralectotype of *B. woodwardi*. (*a*) Paralectotype NYSM 9910. (*b*) Paralectotype NYSM 9911. (*c*) Lectotype NYSM 9909. (*d*) Paralectotype NYSM 9912 (all photos credited to Russell Bicknell). Scale bars = 1 cm.

**Figure 5.**
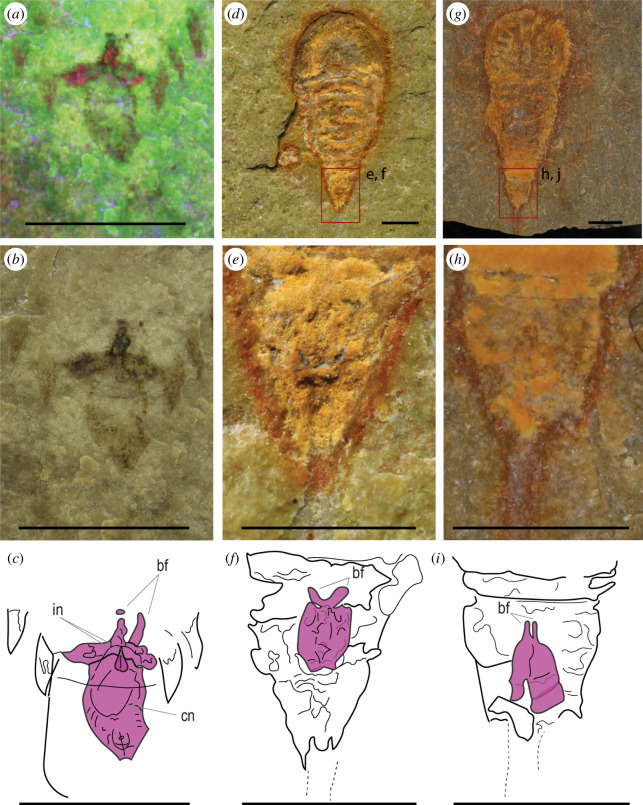
Pretelsonic processes of *B. woodwardi* and *S. abundantis* (Offacolidae). (*a,b*) Multi-spectral imaging (*a*) and optical photography (*b*) close-ups of the pretelsonic process of *B. woodwardi* specimen ROMIP53886 from the boxed areas in figure 1*c,a*, respectively. (*c*) Interpretative line drawing of the pretelsonic process of *B. woodwardi* specimen ROMIP53886 based on (*a,b*). (*d–f*) Habitus view of *S. abundantis* specimen MGL102902 (*d*), and close-up (*e*) and interpretative line drawing (*f*) of its pretelsonic process. (*g–i*) Habitus view of S*. abundantis* specimen YPM IP 517932 (*g*), and close-up (*h*) and interpretative line drawing (*i*) of its pretelsonic process. Abbreviations: in, insertion; bf, bifurcation; cn, centrum. The different pretelsonic processes are highlighted in purple. Scale bars = 1 cm (*a–c*), 1 mm (*d–i*).

. 1920 *Bunaia woodwardi* Clarke, pp. 129, pl. 1, figs. 1–4;

. 1925 *Bunaia woodwardi* Ruedemann, pp. 79, pl. 24, figs. 1–3;

. 1974 *Bunaia woodwardi* Eldredge and Smith, pp. 24, fig. 8;

*2009 specimen ROMIP53886 figured as *Bunaia woodwardi,* Rudkin and Young, pp. 35, fig. 6.

(figures 1–4, 5*a*–*c*).

*Emended diagnosis.* Large offacolid species with a total length of up to 32 mm (telson excluded). Prosoma long, about 30% of the total body length (telson excluded). Thick doublure. Eleven opisthosomal somites. Presence of triangular-shaped pleural spine in opisthosomal somite VII–X. The first opisthosomal tergite is more than four times wider than the 11th tergite, and the ninth tergite is two times wider than the 11th.

*Type and referred material:* Lectotype: NYSM 9909 (figure 4); Paralectotypes: NYSM 9910 (figure 4), NYSM 9911 (figure 4) and NYSM 9912 (figure 4). In Eldredge [42, p. 24], this material is referred as ‘Types. I designate NYSM 9909 as lectotype, and NYSM 9910–9912 as three paratypes’. Although Eldredge referred to the remaining specimens as paratypes, according to current nomenclatural standards they should be referred to as paralectotypes. Therefore, we here update the status of specimens NYSM 9910, 9911 and 9912 to paralectotypes [43]. Other referred material includes ROMIP53886 (part; figure 1*a–d*); ROMIP51447 (part; figure 2*a–c*); ROMIP50689 (part; figure 3*a–c*); ROMIP50690 (part; figure 3*d–f*).

### 3.2. Description

#### 3.2.1. ROMIP53886

ROMIP53886 (figure 1*a*–*d*) is an almost complete specimen that has been dorso-ventrally flattened during preservation. It possesses an obovate-shaped body that is divided into the following three tagma: an anterior prosoma overlapped by a dorsal headshield, and an unfused opisthosoma not clearly differentiated into a pre-abdomen and an abdomen. The total length of the body (from the tip of the prosoma to the last somite) is 32 mm. The prosoma represents the widest region of the body, and its preservation is incomplete on the left side, so the maximum width is estimated to *ca* 22 mm.

The headshield is lunate in outline (13 mm long and 22 mm wide). A small genal spine is present on the posterior-left edge (gs in figure 1*b*). A well-developed doublure completely surrounds the prosomal shield ventrally (db in figure 1*b*). No labrum or ophthalmic region can be observed. Two small flat dots medially on the doublure may represent median sensory organs (mso in figure 1*b*).

The prosoma bears six pairs of appendages inserted around the mouth. The first pair of appendages are uniramous, elongate chelicerae and anteroventrally oriented (grey structures in figure 1*b*). However, there is no evidence that the last podomeres form a chela. Appendages 2–5 are biramous and latero-ventrally oriented (en2−6 and ex2−5 in figure 1*b*). Endopods 2 and 3 are bent and allow the recognition of the associated exopods 2 and 3 (en2−3 and ex2−3 in figure 1*b*). Appendage 6 is possibly uniramous (en6 in figure 1*b*). The endopods are preserved and retracted. Details of the anatomy of the podites are not preserved in both external and internal rami.

The prosoma is long, representing about 30% of the total body length (short opisthosoma measures 19 mm and long prosoma measures 13 mm). The opisthosoma consists of 11 somites (somite VII–XVII). It has a trapezoidal shape with the longest side coinciding with the first opisthosomal tergites (somite VII, 3.3 mm wide) and the short side coinciding with the last somite (somite XVII, 1.5 mm wide). A clear boundary between pre-abdomen and abdomen is absent. However, a discontinuity in the pleural spines (ps in figure 1*b*) occurs from the seventh somite, underlining the probable border between pre-abdomen and abdomen. The first opisthosomal somite (somite VII) appears reduced, partially or fully overlapped by the prosomal shield and no pleurae are noted. The imprint of the axis is present in somite VIII–XII (opisthosomal tergites 2–6), with no sign of antero-posterior bipartite sclerotization noted. No sub-axial node is observed. Tergites 1–6 possess pleura with a square shape. The width of the tergites decreases slightly from tergites 3–10 (from 3.3 mm for tergite 3–1.5 mm for tergite 10). Tergites 1–10 bear pleural spines.

The first opisthosomal somite (somite VII) bears a pair of appendages (ex6 in figure 1*b*), while somite VIII preserves only the proximal part of them. The first opisthosomal appendages are paddle-like uniramous exopods and insert medially to the somite, in the axial region. Traces of the insertion point of the following appendages are also preserved in somites X and XII.

The abdomen is probably composed of four somites (somites XIV–XVII) based on the pleural anatomy; however, the absence of preservation of the dorsal anatomy is an obstacle to an unequivocal description. Triangular pleural spines are present on the sides of the abdominal tergites (ps in figure 1*b*). Somite XVII is three times longer than the previous somite (somite XVI is 1.5 mm long and somite XVII is 4.5 mm long) and devoid of pleural spines.

A symmetrical process in a pretelsonic position is preserved ventrally to somite XVI–XVII (purple structure in figure 1*b*; see also figure 5*a–c*). This pretelsonic process is longitudinally bipartite and has a bifurcated proximal tip distally (bf in figure 5*c*). It is formed by two branches departing from two points at the margin of somite XVI–XVII (in figure 5*c*), diverging into an oval shape and meeting again on the distal end, ventrally to the XVII somite. A terminal telson is present, but not completely preserved.

#### 3.2.2. ROMIP51447

ROMIP51447 (figure 2*a–c*) ventrally preserves only the prosomal region. A well-developed doublure completely surrounds the prosomal shield ventrally. No labrum is observed. The prosoma bears six pairs of appendages that are better preserved on the right side of the specimen. The first pair of uniramous appendages, interpreted as the chelicerae, are retracted. There is no evidence that the last podomeres form a chela. Appendages 2–3 are well preserved, biramous and latero-ventrally oriented. Appendages 4–6 are preserved, but a distinction between external and internal rami is not possible. Anatomical details of the podites are not preserved in both exopodite and endopodite.

#### 3.2.3. ROMIP50689

ROMI50689 (figure 3*a–c*) also ventrally preserves only the prosomal region. A well-developed doublure completely surrounds the prosomal shield ventrally. An apical tip is present at the anterior edge of the prosoma (pt in figure 3*b*), and a small genal spine is visible on the left side (gs in figure 3*b*). No labrum is observed. The prosomal appendages are preserved (ap in figure 3*b*), but the inner and external rami cannot be distinguished owing to preservation quality. In the middle proximal part of the prosoma, the imprint of what could be the median ridge (cardiac ridge) is preserved (mr in figure 3*b*).

#### 3.2.4. ROMIP50690

ROMIP50690 (figure 3*d–f*) partially preserves the prosomal and opisthosomal anatomy. It is dorso-ventrally flattened in its preservation. The dorsal prosomal anatomy is completely missing. A general imprint of the appendages is preserved but lacks all anatomical details (ap in figure 3*e*). The opisthosoma preserves six somites but only in the tergites axial anatomy (ax1−6 in figure 3*e*). In the centre of the opisthosoma, a straight tubular structure runs along the first three segments. It preserves two lobes departing from the left sides and is interpreted as the digestive system (gu in figure 3*e*). No pleura or opisthosomal appendage anatomy is preserved. Abdomen and telson are not preserved.

### 3.3. Phylogenetic analyses results

**Figure 6.**
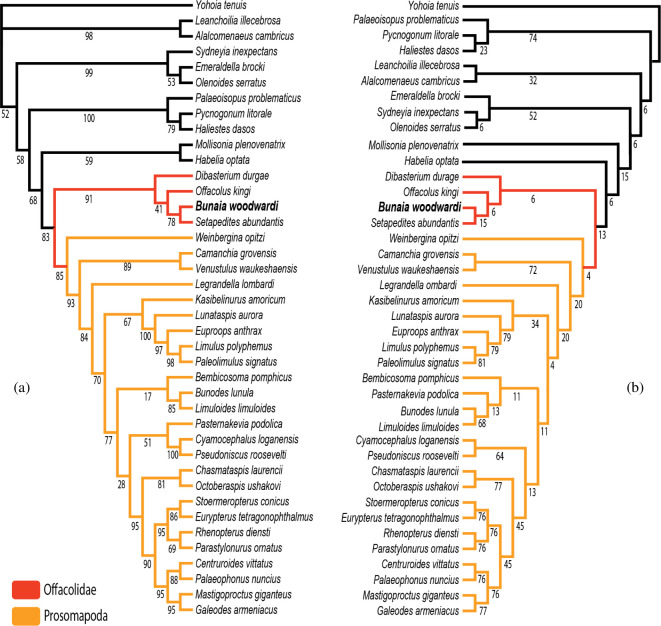
Phylogenetic position of *B. woodwardi* among euchelicerates. (*a*) Maximum clade credibility tree of Bayesian analysis of euchelicerates. Numbers next to nodes are posterior probabilities when less than 100. (*b*) Implied weights *K* = 12 maximum parsimony tree of euchelicerates. Numbers next to nodes are relative Bremer support. Both trees are based on a matrix of 40 taxa and 114 characters modified by Lamsdell [12] and Lustri *et al*. [8].

All phylogenetic analyses conducted for this study using the newly described characters from specimens ROMIP53886, ROMIP51447, ROMIP50689 and ROMIP50690 identified *B. woodwardi* as a member of the family Offacolidae. This was the case using both parsimony (with equal weight, as shown in electronic supplementary material, figure S1, and implied weight with various *K* values, as shown in electronic supplementary material, figures S2–S4 for *K* = 3, *K* = 6 and *K* = 9, and in figure 6*a* for *K* = 12) and Bayesian methods (figure 6*b* for maximum clade credibility tree and electronic supplementary material, figure S5 for 50% majority rule consensus). In all iterations of our phylogenetic analyses, *Setapedites* and *B. woodwardi* are consistently resolved as sister taxa within the Offacolidae clade based on the following synapomorphies: the presence of an anterior projection from the carapace (prosomal shield) and the carapace being longer than wide.

### 3.4. Remarks

#### 3.4.1. Interpretation of the biramous appendages

The interpretation of the appendages of specimen ROMIP53886 as biramous and not uniramous is based on the following three points: (i) both endopod 2 and exopod 2 are visible and depart from the same point; (ii) endopod 3 is bent, not perfectly on the same plane as the matrix, allowing the angle it generates to be seen; (iii) if uniramous, the appendages would depart far from the doublure in a mesial insertion point along the ventral surface, whereas they are often found closer to the doublure in *Weinbergina*, *Dibasterium* and *Offacolus*. This reasoning, along with the clearer biramous anatomy of the less well-preserved appendages of specimen ROMIP51447, supports our interpretation of *B. woodwardi* possessing biramous appendages.

#### 3.4.2. The genus *Bunaia* and its possible synonymy with the genus *Pseudoniscus*

Two Silurian-aged species of the genus *Bunaia* have been previously described. The first is *B. woodwardi* [26] from Bertie Group of East Buffalo (New York State, USA) and the second is *Bunaia heintzi* [44] from Ringerike Sandstone (Norway). The Norwegian material consists of an isolated prosoma, and its assignment to the genus *Bunaia* has previously been questioned. Eldredge & Smith [42] pointed out that the differences in prosomal shape, the larger size and the presence of a marked granulation in *B. heintzi* may justify its allocation to a new genus.

Before this formal description of the four specimens from the ROM, *B. woodwardi* was known only from four poorly preserved specimens [26,42] (figure 4). Specimen ROMIP53886 (figure 1*a–c*) was subsequently figured by Rudkin & Young [45] and referred to as *B. woodwardi* but not officially described. The specimens are here assigned to species *B. woodwardi* based upon their coeval stratigraphical and geographical occurrence, numerous shared characteristics and the absence of inconsistent characters (see §4.1). We also remark that the genus *Bunaia* itself has been questioned as a possible synonym of *Pseudoniscus clarkei* Nieszkowski, 1859 [42]. The genus *Pseudoniscus* includes four species from Europe and North America, nominally *P. aculeatus* from the Oesel group in Estonia [46], *P. falcatus* from the Patrick Burn Formation of Scotland [47], *P. roosevelti* from Pittsford shale of New York State (US) [48] and *P. clarkei* from the Bertie Group of New York State (US) [49]. A broad review of the material assigned to the genus *Pseudoniscus* is required in order to assess whether *Bunaia* should be synonymized with *Pseudoniscus* (in which case *Pseudoniscus* and its family Pseudoniscidae would be senior synonyms, as these names were published first).

## 4. Discussion

### 4.1. Attribution of the newly described material to *Bunaia woodwardi*

The exceptional quality of preservation of the new specimens described herein reveals previously unknown anatomical features in great detail but presents some uncertainty when uniting these specimens (figures 1–3) with the original *B. woodwardi* lectotype and paralectotypes (figure 4) because of the comparably poor preservation of the original type specimens. After the first brief description [26], the four original specimens of *B. woodwardi* (figure 4; NYSM 9909, NYSM 9910, NYSM 9911 and NYSM 9912) were redescribed in 1974 by Eldredge & Smith [42]. This redescription led to the identification of numerous taxonomically significant characteristics including the presence of a small genal spine, a curved posterior margin of the prosoma, the presence of a cardiac ridge and the median sensory organs [42]. Most of these anatomical features are also observed in the specimens housed in the ROM. The small genal spine and curved posterior margin of the prosoma are seen in ROMIP53886, the presence of a cardiac ridge (median ridge) is suggested by ROMI50689 and possible median sensory organs have also been identified in ROMIP53886 (figures 1*a–c* and 3*a–c*). Other characters shared among all the specimens include an expanded doublure (ROMIP51447, ROMIP53886, ROMIP50689 and NYSM 9909, respectively; figures 1–4c) and an opisthosomal axis (ROMIP50690, NYSM 9911, respectively, figures 3*d–f* and 4*b*). These characteristics taken together substantially contribute to uniting these specimens with the species *B. woodwardi*. However, most of these characters are also commonly found in other synziphosurine taxa, e.g. *Legrandella lombardii*, *Bunodes lunula, Cyamocephalus loganensis* and *Weinbergina opitzi* [42,50–52] and as such are of weak broader taxonomical relevance. Other factors support placing the ROM specimens within *B. woodwardi*. Firstly, all the specimens originated from the Bertie Formation, even if from two different localities (Ridgemount Quarry in Ontario and East Buffalo in New York State). The specimens are not disparate in time and space, only in preservation quality. Secondly, none of the original paralectotype specimens preserves traces of appendages, the pretelsonic process or the abdominal pleural spine that contradict what can be identified in the better preserved and newly described material herein [26,42] (figures 1–4). Therefore, it is necessary to balance the shared anatomy, the coeval stratigraphy and the geographical proximity of the fossil sites against the lack of more specific diagnostic characters in the original type material. *Pseudoniscus clarkei* [49], also from the Bertie Group, has been considered for the assignation of the newly described material; however, a revision of the entire genus is required before it is possible to compare it with the material here ascribed as *B. woodwardi*, but their close affinities are noted. On balance, it seems reasonable to assign the new material and its unique features to the existing species *B. woodwardi* while providing an emended diagnosis for the species based on the new better-preserved specimens.

### 4.2. *Bunaia woodwardi* is an offacolid

All phylogenetic analyses conducted under parsimony (using both equal weight; see electronic supplementary material, figure S1, and implied weight with an array of different *K* values, see electronic supplementary material, figures S2–S4 for *K* = 3, *K* = 6, *K* = 9 and figure 6*a* for *K* = 12) and Bayesian methods (figure 6*b* for maximum clade credibility tree and electronic supplementary material, figure S5 for 50% majority rule consensus) retrieved *B. woodwardi* as a member of the family Offacolidae. Prosomal appendages are crucial characters to untangling the sequence of early euchelicerate evolution: the clade Prosomapoda is defined as euchelicerates bearing uniramous prosomal appendages [12], whereas the sister clade of prosomapods, the Offacolidae [15], is defined by the presence of biramous prosomal appendages [12]. These two monophyletic groupings, Prosomapoda and Offacolidae, collectively comprise the monophyletic group of Euchelicerata [8] (figure 6).

The new specimens attributed to *B. woodwardi* (figures 1–3) described herein possess their own suite of diagnostic characters (size, thick doublure, morphology of the abdomen) and also show compelling anatomical affinity to the Offacolidae [8,13–15]. *Bunaia woodwardi* shares three major distinctive characteristics with other Offacolidae. Firstly, the stenopodous nature of exopods of the biramous prosomal appendages [8], an apomorphy that the Offacolidae share with the Cambrian Habeliida [19,28]. Secondly, the elongated uniramous first pair of appendages (chelicerae). Thirdly, the uniramous and paddle-like pair of post-cheliceral appendage 6, apomorphic of the Offacolidae [8]. In addition to these, *B. woodwardi* also shares with *Setapedites*, the Fezouata Offacolidae, a pretelsonic process [8], making this structure another possible apomorphy of the Offacolidae. Other distinctive plesiomorphic characters include the presence of a doublure, a moderate to highly developed axis, the number of appendages in the prosoma and the reduced first tergites, all of which are found preserved in the specimens of *B. woodwardi* (figures 1–3) and suggest this species is a member of Offacolidae. The detailed anatomy of the stenopodous exopods found in *B. woodwardi* is obscure (figures 1 and 2). Further fossil specimens are needed to help clarify the detailed anatomy of this key character for the Offacolidae. As stem-euchelicerates, the presence of chelicerae is considered plesiomorphic for Offacolidae. However, the characteristic elongated chelicerae visible in figure 1 are probably a derived character of offacolids, as supported by our phylogenetic analyses, each iteration of which identifies outgroups without multi-segmented deutocerebral appendages, but instead short appendages [19] and possibly short and robust chelicerae [28]. The elongate morphology and the high number of segments of these chelicerae, as shown in *Dibasterium*, make them morphologically similar to the homologous antenna [13,53,54]. The morphology of the first pair of appendages in the euchelicerates is similar to that found in their possible close relatives, such as the great appendage of the megacheirans [55], the uniramous short appendages of habeliids [19] and the chelifores of pycnogonids [56]. However, as the interrelationships between these major groups remain unclear, it is not yet possible to trace the evolutionary path that led from antenna-like deuterocerebral appendages [53] to the chelate and elongated chelicerae of offacolids. The relationship of offacolids with habeliids [8,19] is sustained in this work, and this suggests that the evolution of the chelicerae occurred earlier in the evolution of Euchelicerata, followed by a diversification early in the group history, with derived forms found in the Prosomapoda and the Offacolidae.

An expanded cephalic doublure is present in *B. woodwardi* (figures 1, 2 and 3*a–c*) as well as in *Setapedites,* which are, respectively, the oldest and youngest representatives of Offacolidae. *Dibasterium* and *Offacolus* do not possess a cephalic doublure [13–15]. The cephalic doublure is considered to be of limited phylogenetic importance as it is plesiomorphic and found in numerous Cambrian arthropods such as *Sidneyia inexpectans* [24,57,58], *Emeraldella brocki* [18,59], aglaspidids [60] and all trilobites [61]. Among Palaeozoic euchelicerates, the doublure is commonly present among Xiphosura [62] and also in synziphosurines [42,51]. The presence of a doublure in the Offacolidae adds value to the interpretation of it as a plesiomorphic condition for the whole of the Euchelicerata, and its loss is derived in *Offacolus* and *Dibasterium,* although a homoplastic evolution of this character in *Setapedites* and *B. woodwardi* after its loss at the root of Offacolidae cannot be excluded.

Finally, when comparing the pretelsonic process of *Setapedites* and *B. woodwardi* (figures 1*a–c* and 5*a–c*), it can be seen that both of these processes are found in the pretelsonic somite and with an attachment point on the border of the previous somite. The shapes are similar, with a horizontal bilateral symmetry expressed by a midline in the middle of the process and a characteristic bifurcation proximal to the insertion point. The newly described pretelsonic process in *B. woodwardi* not only solidifies the interpretation of the same character in the related *Setapedites* but also suggests that the pretelsonic process might be an apomorphic character of the family Offacolidae. The published descriptions of *Offacolus* and *Dibasterium* have not reported the presence of any particular posterior trunk morphology; however, the thin sections-based three-dimensional computerized reconstruction of *Offacolus* specimen OUM C.29557 ([15]; figure 1*a,g*) shows a rounded process on the ventral surface below the pretelsonic tergite and preceding the telson. A re-evaluation of *Offacolus* and *Dibasterium* is needed to establish the nature of this structure and its relation to the pretelsonic process of other offacolids. Otherwise, a secondary loss of this character could also explain its absence in both *Offacolus* and *Dibasterium*.

### 4.3. Nature of the different posterior trunk morphologies and their implication for the Arachnomorpha

#### 4.3.1. Posterior trunk morphology

**Figure 7.**
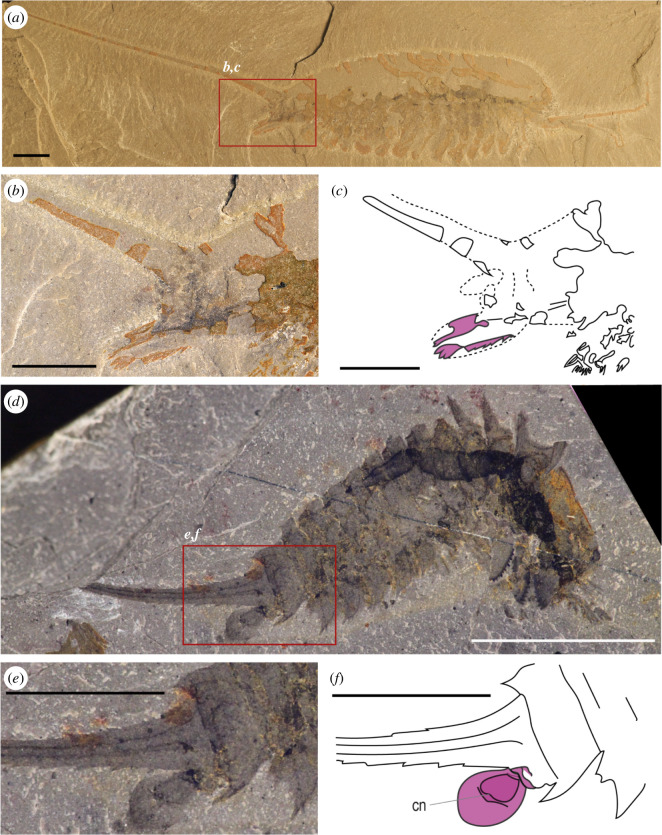
Appendicular derivatives of *Emeraldella brutoni* (Vicissicaudata) and the anal pouch of *H. optata*. (*a–c*) Habitus view of *E. brutoni* specimen UMNH.IP.6162 (*a*), and close-up (*b*) and interpretative line drawing (*c*) of its pretelsonic appendages. (*d–f*) Habitus view of *H. optata* specimen ROMIP64359 (*d*), and close-up (*e*) and interpretative line drawing (*f*) of its anal pouch. Abbreviations: cn, centrum. Appendicular derivatives are highlighted in purple. Photography credits: Javier Ortega-Hernández (*a,b*), (*c*) Royal Ontario Museum, Jean-Bernard Caron. Scale bars = 5 mm.

**Figure 8.**
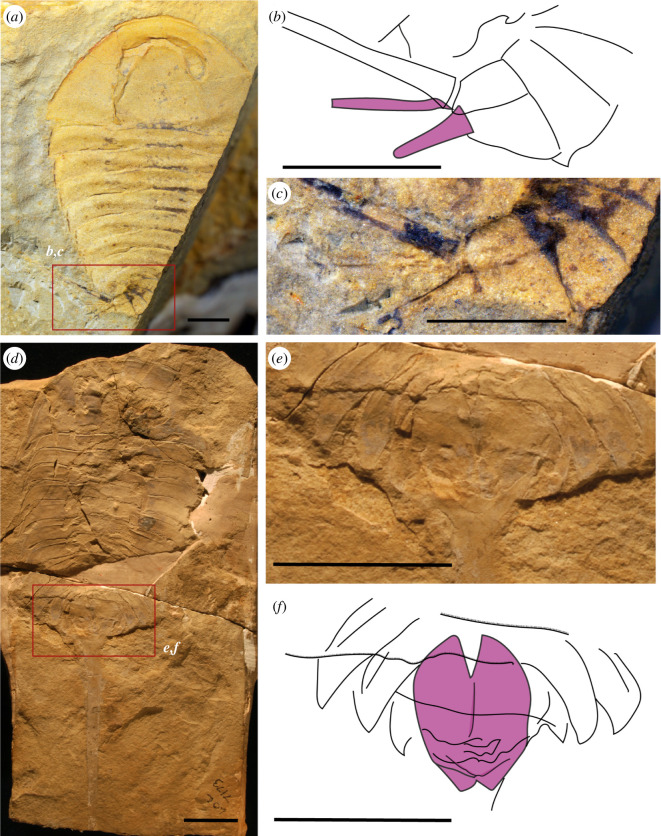
Appendicular derivatives of Aglaspidida. (*a–c*) Habitus view of *Glypharthrus tridentatus* specimen NIGPAS165042a (*a*), and close-up (*b*) and interpretative line drawing (*c*) of its pretelsonic appendages. (*d–f*) Habitus view of *A. spinifer* specimen MPM11155 (*d*), and close-up (*e*) and interpretative line drawing (*f*) of its pretelsonic appendages (post-ventral plate). The different appendicular derivatives are highlighted in purple. Photography credits: Xuejian Zhu (*a,b*), Javier Ortega-Hernández (*d,e*). Scale bar = 1 mm (*a–c*), 2 mm (*d–f*).

Arachnomorpha (*sensu* Størmer [27]) is the monophyletic group of arthropods composed of Artiopoda (*sensu* Hou [30]) and Chelicerata. This group has been recovered in different phylogenetic analyses with different approaches [4,19,29]. Among the Artiopoda, the two main divisions are the Trilobita and the Vicissicaudata [23,36,37,63]. However, the affinity of the Artiopoda with the Chelicerata is not always recovered in phylogenetic analyses [23,64]. In this phylogenetic framework, Vicissicaudata is a monophyletic group of taxa with a variety of differentiated posterior trunk morphologies, united by the presence of appendicular derivatives sharing a homologous origin [23,36,63]. Even though this character can be secondarily lost, as observed in *Carimersa neptuni* [65], the appendicular derivatives are the most distinctive features uniting the group [36]. Vicissicaudata includes the genera *Emeraldella* [18,66] and *Sidneyia* [24], which possess a uropod and caudal flap (figure 7*a–c*); the Aglaspidida [18], which possess a post-ventral plate or a furcal rami (figure 8); and the Cheloniellida [25], which possess furcae [67] (the terminology used for the posterior trunk morphology in this paper and in the literature is summarized in table 1).

**Table 1.** The terminology used to refer to the different posterior trunk morphologies and their appendicular derivatives.

general terminology	clade	specific terminology referring to the appendicular derivatives	reference
	*Sidneya*	uropod or flaps	Ortega‐Hernández *et al*. [23]; Bruton [57]
	*Emeraldella*	uropod or flaps	Ortega‐Hernández *et al*. [23]; Stein & Selden [59]; Stein *et al*. [66]; Lerosey-Aubril & Ortega-Hernández [37]
	Cheloinellida	furcae	Ortega‐Hernández *et al*. [23]; Cotton & Braddy [29]; Chlupác [67]
	Aglaspida	post-ventral plate, pre-telsonic appendages or furcal rami	Ortega‐Hernández *et al*. [23]; Van Roy [68]; Cotton & Braddy [29]; Hesselbo [69]; Lerosey-Aubril *et al*. [36]
	*Habelia*	anal pouch, para-anal lamina (in Italian,‘lamina paraanale’)	Aria & Caron [19]; Simonetta [20]
	Offacolidae	pretelsonic process	Lustri *et al*. [8]

posterior trunk morphology	clade	general terminology of modified appendages	reference
	*Sidneya*	appendicular derivatives	Lerosey-Aubril & Ortega-Hernández [37]; Lerosey- Aubril *et al.* [36]
	*Emeraldella*	appendicular derivatives	Lerosey-Aubril & Ortega-Hernández [37]; Lerosey- Aubril *et al.* [36]
	Cheloinellida	appendicular derivatives	Lerosey-Aubril & Ortega-Hernández [37]; Lerosey- Aubril *et al.* [36]
	Aglaspida	appendicular derivatives	Lerosey-Aubril & Ortega-Hernández [37]; Lerosey- Aubril *et al.* [36]
	*Habelia*	appendicular derivatives	present work
	Offacolidae	appendicular derivatives	present work

To better understand the Vicissicaudata, several studies have tried to establish the homology of the different posterior trunk morphologies, focusing on the appendicular derivatives and last two somites involved [23,29,36], using mainly a positional criterion [70] and acknowledging the fact that all these structures are paired [29,68]. Indeed, those differentiated appendicular derivatives seem to be always located on the last somite of the abdomen, even though in the evolution of the post-ventral plate and the furcal rami of the Aglaspidida an alternative hypothesis has been proposed, with the post-ventral plate possibly originating from the second pretelson somite (tergite 11) and the furcal rami from the pretelsonic somite (tergite 12), meaning they are not homologous [36]. The discovery of *Setapedites* [8] from the lower Ordovician Fezouata Lagerstätte [16,17], however, suggested that the family Offacolidae may share a homologous posterior trunk morphology with the Vicissicaudata, a hypothesis that if confirmed would add strong support to the Arachnomorpha as a monophyletic group. In particular, the pretelsonic process of *Setapedites* (and now *B. woodwardi*) would be homologous to the appendicular derivatives characteristic of the Vicissicaudata posterior trunk morphology (see supplemental discussions in Lustri *et al*. [8]). The anal pouch of *H. optata* (figure 7*d–f*) has also been considered as a possible homologue to the pretelsonic process of *Setapedites* (see supplemental discussions in Lustri *et al*. [8]), because Offacolidae and Habeliida have been recovered as closely related groups in phylogenetic analyses and because of the numerous characters shared between the groups [8,19,28].

**Figure 9.**
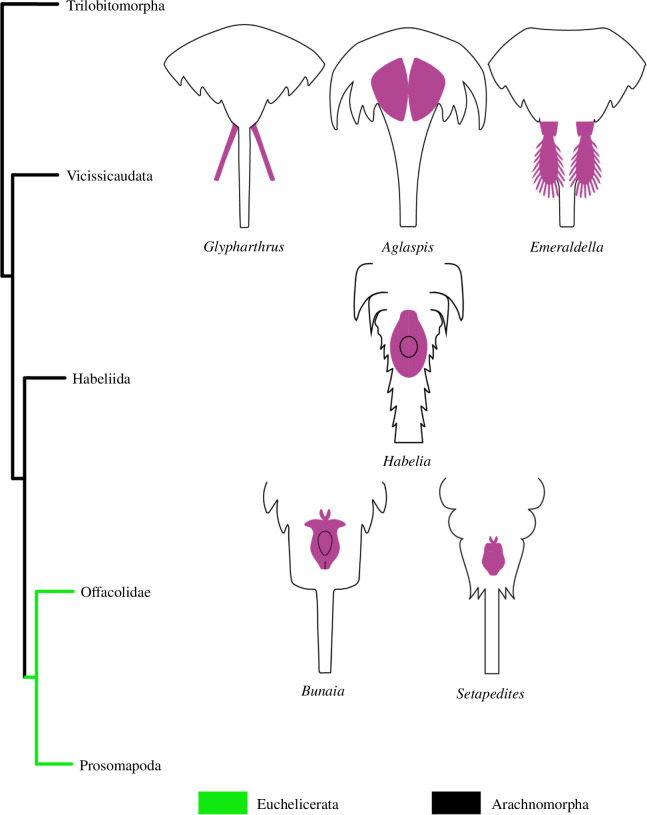
Schematic reconstructions of different posterior trunk morphologies of *Habelia*, Vicissicaudata and Offacolidae plotted onto a hypothetical phylogenetic relationship of Arachnomorpha. Phylogenetic tree based upon the present study results and refs. [4,8,12,36]. Schematic reconstructions drawn from refs. [8,19,23]. The different appendicular derivatives are highlighted in purple.

The following three main interpretations have been suggested (see supplemental discussions in Lustri *et al*. [8]). First, that the pretelsonic process of offacolids is an anal pouch like that in *H. optata*. Second, that the pretelsonic process of offacolids is homologous to the appendicular derivatives characteristic of the Vicissicaudata. And third, if both the previous interpretations are correct, all the supposed appendicular derivatives (*H. optata* anal pouch, Vicissicaudata appendicular derivatives and Offacolidae pretelsonic process) are homologous and reflect the apomorphic state of the inner branches of Arachnomorpha (figure 9). This can be resolved by examining the nature of the posterior trunk morphologies of the groups involved.

#### 4.3.2. Pretelsonic process and its homology with Habeliida and Vicissicaudata posterior trunk morphologies

The new specimens of the offacolid *B. woodwardi* are well preserved in the posterior trunk morphology, which allows comparison of this anatomical character among the Offacolidae, the Habeliida and the Vicissicaudata (mainly Aglaspidida and the genus *Emeraldella*) in greater detail than has been possible previously (figures 6–9). The posterior trunk morphology of the Cheloniellida is herein excluded from the comparison because, although it possesses a pair of furcae, these are located on the dorsal side of the posterior trunk, whereas all the other members show the modified appendages ventrally located [23,36,37,57,60], suggesting the dorsal location is a derived character of Cheloniellida [71]. Furthermore, possible new evidence for both a round anal sclerite and furca in *Parioscorpio venator* complicates the picture of the evolution of posterior trunk morphologies in Cheloniellida [72].

Three main criteria are used to establish homology among different characters: (i) shared development, (ii) traceable ancestry for derived characters, and (iii) association criterion [73]. A developmental pattern regarding the posterior trunk morphology is missing in the whole clade, so the first criterion is excluded from our comparison. Similarly, the uncertainty of the phylogenetic relationships among all the clades of interest makes it difficult to discuss the second criterion. Although the results of our phylogenetic analyses support the hypothesis of a common origin for this character, further studies are needed to fully support the evolutionary scenario illustrated in figure 9. This leaves only the third criterion to consider, the position and symmetry of the different posterior trunk morphologies, to establish homology. However, general morphological considerations such as preservation and overall anatomy are also discussed.

First, the position is considered. The pretelsonic process in *B. woodwardi* is clearly shown in specimen ROMIP53886 (figures 1 and *5a–c*). In *H. optata*, the position of the anal pouch is not completely clear. In figure 7*d–f*, the anal pouch appears to be inserted in the telson head, while specimen ROMIP 64368 shows a more proximal insertion [19]. In the Vicissicaudata, the appendicular derivatives have been associated with the pretelsonic somite or the previous somite if the post-ventral plate is not homologous to all the other appendicular derivatives [36]. In the Offacolidae, the two examples of pretelsonic processes in *Setapedites* and *B. woodwardi* share the same position on the pretelsonic somite (figures 1 and 5). With the exception of the Ordovician aglaspidids, which may have had an independent evolution of the post-ventral plate, the position of all the other appendicular derivatives, including *H. optata* and offacolids, support a shared evolution.

Secondly, the bilaterally symmetrical nature of the pretelsonic process is also clear in these Offacolidae and this helps to resolve the position of the homologues with respect to the rest of the anatomy. The double insertion of the *B. woodwardi* pretelsonic process (figure 5*a–c*) probably represents its vestigial paired appendicular nature. An appendicular nature for the pretelsonic process in *B. woodwardi* is also strongly suggested by the fact that its response under multi-spectral imaging is highly consistent with that of the prosomal appendages and different from the rest of the cuticular features. The modified appendicular derivatives of Aglaspidida, *S. inexpectans* and the genus *Emeraldella* also have a bilateral symmetry. This strongly supports the hypothesis of the pretelsonic process having derived from a pair of appendages inserted in the last abdominal segment and being subsequently fused into one single flap. This model is compatible with the evolution of the appendicular derivatives especially in the Aglaspidida, which similarly shows the two plates attached by a less sclerotized membrane [69]. The homology between the pretelsonic process of Offacolidae and the anal pouch of *Habelia* is more difficult to establish in the context of bilateral symmetry. While Simonetta [20] interpreted the posterior trunk morphology of *H. optata* as a ‘para-anal lamina’ closely related to the post-ventral plate of the Aglaspidida, more recently this process was reinterpreted as an anal pouch that is possibly related to sexual dimorphism [19]. However, in the anal pouch model, no possible homologous features in related taxa were identified. Further study on the origins and affinities of the anal pouch in *H. optata* is required; however, it currently cannot be excluded that it originated from appendages that subsequently fused into a single unit.

A general consideration of the preservational differences in the pretelsonic processes is also useful in establishing a homologous origin. In *B. woodwardi*, the pretelsonic process is not a mineralized or rigid plate, as shown by its lack of relief, its indistinct margins, and its irregular surface texture (figures 1 and 5*a–c*), suggesting at most light sclerotization. In *Setapedites,* the rare preservation of the pretelsonic process, seen in only five specimens out of many hundreds, suggests a weakly sclerotized or even membranous nature for this structure [8]. This is probably also the case for the habeliids [19] (figure 7*d–f*), and some of the Vicissicaudata, such as *Emeraldella brutoni* (figure 7*a–c*), which has a caudal flap of the same shape and with the same preservation as the exopods [37]. However, the posterior ventral plate of the aglaspidids is a sclerite [36,74] with a higher degree of sclerotization, as shown by the relief of the structure, its solid margins and its regular surface texture (figure 8). This suggests that relatively light sclerotization could be plesiomorphic for all the appendicular derivatives, with the high degree of sclerotization in the posterior ventral plate of aglaspidids being a derived state among all the appendicular derivatives.

Finally, the flap-like anatomy of the pretelsonic process in *B. woodwardi* can be recognized in the anal pouch of *Habelia*, in that both share a similar rounded or sub-oval shape and a characteristic rounded centrum (see figure 5, 7*d*–*f* and 9 for comparison). Other appendicular derivatives do not show a similar sub-oval shape and a rounded centrum. The close affinities of *H. optata* and offacolids [8,19] explain this unique combination of characters in their appendicular derivatives as apomorphic traits for *H. optata* and Offacolidae.

## 5. Conclusion

New anatomical data described in *B. woodwardi* allow for the placement of this taxon within the family Offacolidae and solidifies the status of this family as a monophyletic group of stem-euchelicerates. The diversity of Offacolidae is now known to be higher, with greater disparity of the abdomen relative size and morphology, and the temporal range of the clade has been extended (by almost 15 Myr) to the end of the Silurian. Our re-evaluation of synziphosurine taxa using new methodologies (multi-spectral imaging) allows for a more detailed description of the ventral anatomy. Considering the position and symmetry as well as the general morphology, the appendicular derivatives in the Vicissicaudata and *H. optata* are probably homologous to the pretelsonic process in the Offacolidae, meaning that these taxa share a homologous posterior trunk morphology. However, the lack of a fully resolved phylogenetic framework remains problematic when attempting to establish homology. Further study on the anal pouch of *H. optata* and new phylogenetic studies accounting for these characters would confirm or deny whether posterior trunk morphologies in the Vicissicaudata and Offacolidae share an ancient appendicular origin. A possible phylogenetic framework with graphical schematic reconstructing of all the discussed appendicular derivatives is presented in figure 9.

The anatomical analyses of posterior trunk morphologies herein support the Arachnomorpha as a monophyletic group that includes Euchelicerata, Vicissicaudata and Habeliida and help to resolve the highly enigmatic phylogeny at the base of the euchelicerates. This has resulted in better resolution in the euchelicerate stem lineage and the sequence of character acquisition during early euchelicerate evolution.

## Data Availability

Requests for access to the four new specimens of Bunaia woodwardi described herein should be addressed to Jean-Bernard Caron (jcaron@rom.on.ca) of the Royal Ontario Museum (Toronto, Canada). All photographs and multi-spectral images generated in the course of this project, as well as the matrices and code necessary to run the phylogenetic analyses, are freely available on SWISSUbase [75]. Supplementary material is available online [76].

## References

[1] Giribet G. 2018 Current views on chelicerate phylogeny—a tribute to Peter Weygoldt. Zool. Anz. **273**, 7–13. (10.1016/j.jcz.2018.01.004)

[2] Giribet G, Edgecombe GD. 2019 The phylogeny and evolutionary history of arthropods. Curr. Biol. **29**, 592–602. (10.1016/j.cub.2019.04.057) 31211983

[3] Edgecombe GD. 2010 Arthropod phylogeny: an overview from the perspectives of morphology, molecular data and the fossil record. Arthropod Struct. Dev. **39**, 74–87. (10.1016/j.asd.2009.10.002) 19854297

[4] Legg DA, Sutton MD, Edgecombe GD. 2013 Arthropod fossil data increase congruence of morphological and molecular phylogenies. Nat. Commun. **4**, 1–7. (10.1038/ncomms3485) 24077329

[5] Wheeler WC, Hayashi CY. 1998 The phylogeny of the extant chelicerate orders. Cladistics **14**, 173–192. (10.1111/j.1096-0031.1998.tb00331.x) 34902927

[6] Dunlop JA, Arango CP. 2005 Pycnogonid affinities: a review. J. Zool. Syst. **43**, 8–21. (10.1111/j.1439-0469.2004.00284.x)

[7] Dunlop JA. 2010 Geological history and phylogeny of chelicerata. Arthropod Struct. Dev. **39**, 124–142. (10.1016/j.asd.2010.01.003) 20093195

[8] Lustri L, Gueriau P, Daley AC. 2024 Lower Ordovician synziphosurine reveals early euchelicerate diversity and evolution. Nat. Commun. **15**, 3808. (10.1038/s41467-024-48013-w) 38714651 PMC11076625

[9] Moore RA, McKENZIE SC, Lieberman BS. 2007 A carboniferous synziphosurine (Xiphosura) from the Bear Gulch Limestone. Palaeontology **50**, 1013–1019. (10.1111/j.1475-4983.2007.00685.x)

[10] Zittel KV. 1885 Handbuch der palaeontologie. In Palaeozoolgie, vol. 2. Munich, Germany: R. Oldenbourg.

[11] Anderson LI, Selden PA. 1997 Opisthosomal fusion and phylogeny of Palaeozoic Xiphosura. Lethaia **30**, 19–31. (10.1111/j.1502-3931.1997.tb00440.x)

[12] Lamsdell JC. 2013 Revised systematics of Palaeozoic ‘horseshoe crabs’ and the myth of monophyletic Xiphosura. Zool. J. Linn. Soc. **167**, 1–27. (10.1111/j.1096-3642.2012.00874.x)

[13] Briggs DEG, Siveter DJ, Siveter DJ, Sutton MD, Garwood RJ, Legg D. 2012 Silurian horseshoe crab illuminates the evolution of arthropod limbs. Proc. Natl Acad. Sci. USA **109**, 15 702–15 705. (10.1073/pnas.1205875109) 22967511 PMC3465403

[14] Orr PJ, Siveter DJ, Briggs DE, Siveter DJ, Sutton MD. 2000 A new arthropod from the Silurian Konservat-Lagerstätte of Herefordshire, UK. Proc. R. Soc. Lond. B **267**, 1497–1504. (10.1098/rspb.2000.1170) PMC1690702 11007324

[15] Sutton MD, Briggs DEG, Siveter DJ, Orr PJ. 2002 The arthropod Offacolus kingi (Chelicerata) from the Silurian of Herefordshire, England: computer based morphological reconstructions and phylogenetic affinities. Proc. R. Soc. Lond. B **269**, 1195–1203. (10.1098/rspb.2002.1986) PMC1691018 12065034

[16] Van Roy P, Briggs DE, Gaines RR. 2015 The Fezouata fossils of Morocco; an extraordinary record of marine life in the Early Ordovician. J. Geol. Soc. London **172**, 541–549. (10.1144/jgs2015-01)

[17] Van Roy P, Orr PJ, Botting JP, Muir LA, Vinther J, Lefebvre B, el Hariri K, Briggs DEG. 2010 Ordovician faunas of burgess shale type. Nat. New Biol. **465**, 215–218. (10.1038/nature09038) 20463737

[18] Walcott CD. 1912 Cambrian geology and paleontology II. Middle Cambrian Branchiopoda, Malacostraca, Trilobita and Merostomata. Smith. Misc. Coll. **57**, 145–228.

[19] Aria C, Caron JB. 2017 Mandibulate convergence in an armoured cambrian stem chelicerate. BMC Evol. Biol. **17**, 261. (10.1186/s12862-017-1088-7) 29262772 PMC5738823

[20] Simonetta A. 1964 Osservazioni sugli artropodi non trilobiti della Burgess Shale (Cambriano medio). In III contributo. I generi Molaria, Habelia, Emeraldella, Parahabelia (Nov.) Emeraldoides (Nov.). Monitore zool. ital, pp. 215–231, vol. 72.

[21] Raasch GO. 1939 Cambrian merostomata. Special Papers. Geol. Soc. Am. **19**, 1–146.

[22] Whittington HB. 1981 Rare arthropods from the Burgess Shale, Middle Cambrian, British Columbia. Phil. Trans. R. Soc. Lond. B **292**, 329–357. (10.1098/rstb.1981.0033)

[23] Ortega-Hernández J, Legg DA, Braddy SJ. 2013 The phylogeny of aglaspidid arthropods and the internal relationships within artiopoda. Cladistics **29**, 15–45. (10.1111/j.1096-0031.2012.00413.x) 34814371

[24] Walcott CD. 1911 Cambrian geology and paleontology II middle cambrian branchiopoda. In Smithsonian miscellaneous collections, pp. 17–40, vol. **57**.

[25] Broili F. 1932 Eine neue crustacee aus dem rheinischen unterdevon. In Sitzungsberichte der mathematisch-naturwissenschaftlichen (abteilung) klasse der bayerischen akademie der wissenschaften zu munchen, pp. 27–38.

[26] Clarke JM. 1919 Bunaia Woodwardi, a new Merostome from the Silurian Waterlimes of New York. Geol. Mag. **6**, 531–533. (10.1017/S0016756800202100)

[27] Størmer L. 1944 On the relationships and phylogeny of fossil and recent Arachnomorpha: a comparative study on Arachnida, Xiphosura, Eurypterida, Tribolita, and other fossil Arthropoda. Skrifter Utgitt av Det Norske Videnskaps-Academi I Oslo. I. Mat. Nat. Klasse. **5**, 1–158.

[28] Aria C, Caron JB. 2019 A middle Cambrian arthropod with chelicerae and proto-book gills. Nature **573**, 586–589. (10.1038/s41586-019-1525-4) 31511691

[29] Cotton TJ, Braddy SJ. 2003 The phylogeny of arachnomorph arthropods and the origin of the Chelicerata. Trans. R. Soc. Edinb. Earth Sci. **94**, 169–193. (10.1017/S0263593300000596)

[30] Hou XG. 1997 Arthropods of the lower Cambrian Chengjiang fauna, southwest China. Foss. Strata. **45**, 1–116.

[31] Caron JB, Rudkin DM, Milliken S. 2004 A new late Silurian (Pridolian) naraoiid (Euarthropoda: Nektaspida) from the Bertie Formation of southern Ontario, Canada delayed fallout from the Cambrian explosion. J. Paleontol. **78**, 1138–1145. (10.1666/0022-3360(2004)078<1138:ANLSPN>2.0.CO;2)

[32] Brett CE. 1998 Silurian-Early Devonian sequence stratigraphy, cycles and paleoenvironments of the Niagara Peninsula area of Ontario, Canada. Can. Geol. Surv. GSA Ann. Meet. Field Trip Guide 1–30.

[33] Ciurca Jr, S. J. 1990 Eurypterid biofacies of the Silurian–Devonian evaporite sequence: Niagara peninsula, Ontario, Canada and New York. In New York State Geological Association 62nd Annual Meeting, pp. 1–30. Fredonia, New York.

[34] Ciurca Jr, S. J. 1978 Eurypterid horizons and the stratigraphy of Upper Silurian and Lower Devonian rocks of central-eastern New York State. In New York State Geological Association 50th Annual Meeting, pp. 225–249. Syracuse, New York.

[35] Cramer BD *et al*. 2011 Revised correlation of Silurian Provincial Series of North America with global and regional chronostratigraphic units and δ^13^C_carb_ chemostratigraphy. Lethaia **44**, 185–202. (10.1111/j.1502-3931.2010.00234.x)

[36] Lerosey-Aubril R, Zhu X, Ortega-Hernández J. 2017 The Vicissicaudata revisited – insights from a new aglaspidid arthropod with caudal appendages from the Furongian of China. Sci. Rep. **7**, 11117. (10.1038/s41598-017-11610-5) 28894246 PMC5593897

[37] Lerosey-Aubril R, Ortega-Hernández J. 2019 Appendicular anatomy of the artiopod Emeraldella brutoni from the middle Cambrian (Drumian) of western Utah. PeerJ **7**, e7945. (10.7717/peerj.7945) 31687274 PMC6825744

[38] Brayard A, Gueriau P, Thoury M, Escarguel G. 2019 Glow in the dark: use of synchrotron μXRF trace elemental mapping and multispectral macro-imaging on fossils from the Paris Biota (Bear Lake County, Idaho, USA). Geobios. **54**, 71–79. (10.1016/j.geobios.2019.04.008)

[39] Robin N, Gueriau P, Luque J, Jarvis D, Daley AC, Vonk R. 2021 The oldest peracarid crustacean reveals a Late Devonian freshwater colonization by isopod relatives. Biol. Lett. **17**, 20210226. (10.1098/rsbl.2021.0226) 34129798 PMC8205522

[40] Klug C, Landman NH, Fuchs D, Mapes RH, Pohle A, Guériau P, Reguer S, Hoffmann R. 2019 Anatomy and evolution of the first Coleoidea in the Carboniferous. Commun. Biol. **2**, 280. (10.1038/s42003-019-0523-2) 31372519 PMC6668408

[41] Lewis PO. 2001 A likelihood approach to estimating phylogeny from discrete morphological character data. Syst. Biol. **50**, 913–925. (10.1080/106351501753462876) 12116640

[42] Eldredge N, Smith L. 1974 Revision of the suborder Synziphosurina (Chelicerata, Merostomata): with remarks on merostome phylogeny. Am. Mus. Nov. 1–41.

[43] International Commission on Zoological Nomenclature. 1999 International code of zoological nomenclature, 4th edn. London, UK: International Commission on Zoological Nomenclature. 10.3897/zookeys.931.51583 PMC7205856 32405237

[44] Størmer L. 1934 Merostomata from the downtonian sandstones of Ringerike, Norway. Skrifter utgitt av Det Norske Videnskaps-Akademi I Oslo. I. Matem. Naturvid. **1933**, 1–125.

[45] Rudkin DM, Young GA. 2009 Horseshoe crabs – an ancient ancestry revealed. In Biology and conservation of horseshoe crabs (eds JT Tanacredi, ML Botton, DR Smith), pp. 25–44. New York, NY: Springer. (10.1007/978-0-387-89959-6_2)

[46] Nieszkowski J. 1858 Zusätze zur Monographie der Trilobiten der Ostseeprovinzen: nebst der Beschreibung einiger neuen obersilurischen Crustaceen. Arch. für Naturkunde, Liv.-Est. und Kurl. **1**, 345–384.

[47] Woodward H. 1868 I.—On a New Limuloid Crustacean [Neolimulus falcatus] from the Upper Silurian of Lesmahagow, Lanarkshire. Geol. Mag. **5**, 1–3. (10.1017/S0016756800207139)

[48] Clarke JM. 1902 Notes on Paleozoic crustaceans. N.Y. State Mus. Rep. **54**, 83–110.

[49] Ruedemann R. 1916 Account of some new or little-known species of fossils, mostly from the Paleozoic rocks of New York. N.Y. S. M. Bull. **189**, 7–112.

[50] Anderson LI. 1999 A new specimen of the Silurian synziphosurine arthropod Cyamocephalus. Proc. Geol. Assoc. **110**, 211–216. (10.1016/S0016-7878(99)80071-6)

[51] Moore RA, Briggs DEG, Braddy SJ, Anderson LI, Mikulic DG, Kluessendorf J. 2005 A new synziphosurine (chelicerata: Xiphosura) from the late llandovery (silurian) waukesha lagerstätte, wisconsin, usa. J. Paleontol. **79**, 242–250. (10.1666/0022-3360(2005)079<0242:ANSCXF>2.0.CO;2)

[52] Stürmer W, Bergström J. 1981 Weinbergina, a xiphosuran arthropod from the devonian hunsrück slate. Paläontol. Z. **55**, 237–255. (10.1007/BF02988142)

[53] Ortega-Hernández J, Janssen R, Budd GE. 2017 Origin and evolution of the panarthropod head a palaeobiological and developmental perspective. Arthropod Struct. Dev. **46**, 354–379. (10.1016/j.asd.2016.10.011) 27989966

[54] Budd GE. 2021 The origin and evolution of the euarthropod labrum. Arthropod Struct. Dev. **62**, 101048. (10.1016/j.asd.2021.101048) 33862532

[55] Haug JT, Briggs DE, Haug C. 2012 Morphology and function in the Cambrian Burgess Shale megacheiran arthropod Leanchoilia superlata and the application of a descriptive matrix. BMC Evol. Biol. **12**, 1–20. (10.1186/1471-2148-12-162) 22935076 PMC3468406

[56] Brenneis G, Ungerer P, Scholtz G. 2008 The chelifores of sea spiders (Arthropoda, Pycnogonida) are the appendages of the deutocerebral segment. Evol. Dev. **10**, 717–724. (10.1111/j.1525-142X.2008.00285.x) 19021742

[57] Bruton DL. 1981 The arthropod Sidneyia inexpectans, Middle Cambrian, Burgess Shale, British Columbia. Phil. Trans. R. Soc. Lond. B. **295**, 619–653. (10.1098/rstb.1981.0164)

[58] Stein M. 2013 Cephalic and appendage morphology of the Cambrian arthropod Sidneyia inexpectans. Zool. Anz. - A J. Comp. Zool. **253**, 164–178. (10.1016/j.jcz.2013.05.001)

[59] Stein M, Selden PA. 2012 A restudy of the Burgess Shale (Cambrian) arthropod Emeraldella brocki and reassessment of its affinities. J. Syst. Palaeontol. **10**, 361–383. (10.1080/14772019.2011.566634)

[60] Lerosey-Aubril R, Ortega-Hernández J, Zhu X. 2013 The first aglaspidid sensu stricto from the Cambrian of China (Sandu Formation, Guangxi) . Geol. Mag. **150**, 565–571. (10.1017/S0016756812001045)

[61] Whittington H *et al*. 1997 Treatise on invertebrate paleontology, part O, arthropoda 1, trilobita, revised. In Geological Society of America, Boulder, vol. 530. Lawrence, KS: CO and University of Kansas.

[62] Ewington D, Clarke M, Banks M. 1989 A late Permian fossil horseshoe crab (Paleolimulus: Xiphosura) from Poatina, Great Western Tiers, Tasmania. Pap. Proc. R. Soc. Tasmania **123**, 127–131. (10.26749/rstpp.123.127)

[63] Lerosey-Aubril R, Paterson JR, Gibb S, Chatterton BDE. 2017 Exceptionally-preserved late Cambrian fossils from the McKay Group (British Columbia, Canada) and the evolution of tagmosis in aglaspidid arthropods. Gondwana Res. **42**, 264–279. (10.1016/j.gr.2016.10.013)

[64] Edgecombe GD, García-Bellido DC, Paterson JR. 2011 A new leanchoiliid megacheiran arthropod from the lower Cambrian Emu Bay Shale, South Australia. Acta Palaeontol. Pol. **56**, 385–400. (10.4202/app.2010.0080)

[65] Briggs DEG, Siveter DJ, Siveter DJ, Sutton MD, Legg D, Lamsdell JC. 2023 A vicissicaudatan arthropod from the Silurian Herefordshire Lagerstätte, UK. R. Soc. Open Sci. **10**, 230661. (10.1098/rsos.230661) 37538743 PMC10394423

[66] Stein M, Church SB, Robison RA. 2011 A new Cambrian arthropod, Emeraldella brutoni, from Utah. Paleontol. Contrib. **2011**, 1–9. (10.17161/PC.1808.8086)

[67] Chlupác I. 1988 The enigmatic arthropod Duslia from the Ordovician of Czechoslovakia. Palaeontology **31**, 611–620.

[68] Van Roy P. 2005 An aglaspidid arthropod from the Upper Ordovician of Morocco with remarks on the affinities and limitations of Aglaspidida. Earth Environ. Sci. Trans. R. Soc. Edinb. **96**, 327–350. (10.1017/S0263593300001334)

[69] Hesselbo SP. 1992 Aglaspidida (Arthropoda) from the upper Cambrian of Wisconsin. J. Paleontol. **66**, 885–923. (10.1017/S0022336000021016)

[70] Riedl R. 1978 Order in living organisms: a systems analysis of evolution. New York, NY: John Wiley & Sons.

[71] Van Roy P. 2006 Non-trilobite arthropods from the Ordovician of Morocco. Ghent, Belgium: Ghent University.

[72] Braddy SJ, Dunlop JA. 2021 A sting in the tale of Parioscorpio venator from the Silurian of Wisconsin: is it a cheloniellid arthropod? Lethaia **54**, 603–609. (10.1111/let.12457)

[73] Patterson C. 1982 Morphological characters and homology. In Problems of phylogenetic reconstruction (eds KA Joysey, AE Friday), pp. 21–74. London, UK: Academic Press.

[74] Ortega-Hernández J, Van Roy P, Lerosey-Aubril R. 2016 A new aglaspidid euarthropod with a six-segmented trunk from the Lower Ordovician Fezouata Konservat-Lagerstätte, Morocco. Geol. Mag. **153**, 524–536. (10.1017/S0016756815000710)

[75] Lustri L, Gueriau P, Daley A. 2024 Data from: New specimens of Bunaia woodwardi Clarke, 1919 (Euchelicerata): a new member of Offacolidae providing insight supporting the Arachnomorpha (Version 1.0.0) [Dataset]. UNIL data service. (10.48657/j60z-py52) PMC11524597 39479250

[76] Lustri L, Antcliffe J, Gueriau P, Daley A. 2024 Data from: New specimens of Bunaia woodwardi Clarke, 1919 (Euchelicerata): A new member of Offacolidae providing insight supporting the Arachnomorpha. Figshare. (10.6084/m9.figshare.c.7516504) PMC11524597 39479250

